# Supplementary material for: Relationship between Down-Regulation of Copper-Related Genes and Decreased Ferroportin Protein Level in the Duodenum of Iron-Deficient Piglets
Source: Nutrients. 2020 Dec 30;13(1):104. doi: 10.3390/nu13010104 (PMC7823587; doi:10.3390/nu13010104)
Supplement: Supplementary file 1 [file nutrients-13-00104-s001.pdf]

Table S1. Primers for RT-qPCR

Gene	Primer sequence 5' – 3'	
	Forward	Reverse
Hprt	GGCCATCACATCGTAGCCCT	TCGCCCGTTGACTGGTCATT
Sdha	TGTACGGAAGGTCTCTGCGG	CTCCACGACACTCCCCGTTT
18S	AGGAAAGCAGACATCGACCT	ACCTGGCTGTACTTCCCATC
Tbp	AACATGGTGGGGAGCTGTGA	TCTGCTCTGACTTTAGCACCTGTT
Fpn	TCGCCTAGTGTCATGACCAG	CAGAAACACAGACACCGCAA
Atp7b	TGGGATCGGCTGCAAAGTCA	GGCCGCATCTGTTTCTGAGG
Cybrd1	ACCCTCATTTGGGTCCTCCAC	TATACGATGATGGCGATGCCCTG
Hamp	ATCCCAGACAAGACAGCTCACA	TCTTGCAGCACATCCCACAGA
Erfe	CTGCCCTCCCGTCGGAAC	GTCACTCTGCCGGACGAAGA
Dmt1	GCAGTCCCCATAGTGACCTT	AGGTTCCTGTCATGGTGGAG
Atp7a	TGGTGGGTACAGGAGTAGGT	GCCAGGATCTTACTGCGTGAT
Heph	TCGCATCTTTCCAGCCACCT	GTGCTTGCATGCCACCTTTC
Ctr1	TACAGCTGGAGAAATGGCTGGA	AGGCCCTCTCGGGCTATCTT
Epo	GGAGCCCAGAAGGAAGCCATC	GGGAGCACCAAGTCACCTGT

Table S2. List of antibodies used for Western blot and blastp analysis for examined proteins.

Target protein	Primary Ab	Dilution	Epitop /sus scrofa protein seq. alignments (% of identity)	Secondary Ab	Dilution
**Sod1**	Rabbit polyclonal, Abcam Cat# ab16831, RRID:AB_302535	1:2,000	82%	Goat anti-rabbit, polyclonal, Sigma-Aldrich Cat# A6154, RRID:AB_258284	1:20,000
**Fpn**	Affinity purified rabbit anti-mouse Fpn, kind gift from *F.Canonne-Hergaux*, France	1:500	89%	Goat anti-rabbit, polyclonal, Cell Signaling Technology Cat# 7074, RRID:AB_2099233	1:5000
**Heph**	Mouse monoclonal, Santa Cruz Biotechnology Cat# sc-365365, RRID:AB_10841577	1:1,000	89%	Goat anti-mouse, polyclonal, Sigma-Aldrich Cat# A5278, RRID:AB_258232	1:20,000
**L-Ft**	Rabbit polyclonal, kind gift from *P. Santambrogio*, Italy	1:500	81%	Goat anti-rabbit, polyclonal, Sigma-Aldrich Cat# A6154, RRID:AB_258284	1:20,000
**β-actin**	Goat polyclonal, Santa Cruz Biotechnology Cat# sc-1615, RRID:AB_630835	1:2,000	100%	Donkey anti-goat, polyclonal, Santa Cruz Biotechnology Cat# sc-2020, RRID:AB_631728	1:20,000
**Cp**	Antiserum, Goat-anti human, Sigma-Aldrich Cat# C0911, RRID:AB_258717	1:1,000	87%	Donkey anti-goat, polyclonal, Santa Cruz Biotechnology Cat# sc-2020, RRID:AB_631728	1:20,000
**Ccs**	Rabbit polyclonal,	1:1,000		Goat anti-rabbit,	1:20,000

	Santa Cruz Biotechnology Cat# sc-20141, RRID:AB_2073504		88%	polyclonal, Sigma-Aldrich Cat# A6154, RRID:AB_258284	
**Albumin**	Rabbit polyclonal, Santa Cruz Biotechnology Cat# sc-50536, RRID:AB_2226074	1:1,000	70%	Goat anti-rabbit, polyclonal, Sigma-Aldrich Cat# A6154, RRID:AB_258284	1:20,000
**Smad4**	Rabbit Polyclonal, Cohesion Biosciences Cat# CPA1709	1:500	98%	Goat anti-rabbit, polyclonal, Sigma-Aldrich Cat# A6154, RRID:AB_258284	1:10,000
